# *Streptomyces benahoarensis* sp. nov. Isolated From a Lava Tube of La Palma, Canary Islands, Spain

**DOI:** 10.3389/fmicb.2022.907816

**Published:** 2022-05-16

**Authors:** Jose L. Gonzalez-Pimentel, Bernardo Hermosin, Cesareo Saiz-Jimenez, Valme Jurado

**Affiliations:** ^1^HERCULES Laboratory, Evora University, Evora, Portugal; ^2^Instituto de Recursos Naturales y Agrobiologia, Consejo Superior de Investigaciones Cientificas (IRNAS-CSIC), Sevilla, Spain

**Keywords:** *Streptomyces benahoarensis*, lava tube, polyphasic taxonomy, resistome, antimicrobials

## Abstract

Two *Streptomyces* strains, labeled as MZ03-37^T^ and MZ03-48, were isolated from two different samples, a mucolite-type speleothem and a microbial mat on the walls of a lava tube from La Palma Island (Canary Islands). Phylogenetic analysis based on concatenated sequences of six housekeeping genes indicated that both strains belonged to the same species. The closest relatives for both strains were *Streptomyces palmae* CMU-AB204^T^ (98.71%), *Streptomyces catenulae* NRRL B-2342^T^ (98.35%), and *Streptomyces ramulosus* NRRL B-2714^T^ (98.35%). Multi-locus sequence analysis (MLSA), based on five house-keeping gene alleles (i.e., *atpD, gyrB, recA, rpoB*, and *trpB*), indicated that both isolated strains were closely related to *S. catenulae* NRRL B-2342^T^. Whole-genome average nucleotide identity (ANI) scores of both strains were in the threshold value for species delineation with the closest species. Both strains presented a G+C content of 72.1 mol%. MZ03-37^T^ was light brown in substrate and white in aerial mycelium, whereas MZ03-48 developed a black aerial and substrate mycelium. No pigment diffusion was observed in both strains. They grew at 10°C−37°C (optimum 28°C−32°C) and in the presence of up to 15% (w/v) NaCl. MZ03-37^T^ grew at pH 5–10 (optimal 6–9), whereas MZ03-48 grew at pH 4–11 (optimal 5–10). LL-Diaminopimelic acid was the main diamino acid identified. The predominant fatty acids in both strains were iso-C_16:0_, anteiso-C_15:0_, C_16:0_, and iso-C_14:0_. The major isoprenoid quinones were MK-9(H6) and MK-9(H8), and the main polar lipids were aminolipid, phospholipid, and phosphoglycolipid. *In silico* analyses for functional annotation predicted the presence of gene clusters involved in resistome mechanisms and in the synthesis of described antimicrobials such as linocin-M18 and curamycin, as well as different genes likely involved in mechanisms for active compound synthesis, both already described and not discovered so far. On the basis of their phylogenetic relatedness and their phenotypic and genotypic features, the strains MZ03-37^T^ and MZ03-48 represented a novel species within the genus *Streptomyces*, for which the name *Streptomyces benahoarensis* sp. nov. is proposed. The type strain is MZ03-37^T^ (= CECT 9805 = DSMZ 8002); and MZ03-48 (= CECT 9806 = DSMZ 8011) is a reference strain.

## Introduction

The genus *Streptomyces* was originally proposed by Waksman and Hinrici (1943). It is the most representative genus within the phylum *Actinobacteria* with more than 800 described species and subspecies (http://www.bacterio.net/streptomyces.html). Besides, the genus *Streptomyces* constitutes roughly 5% of all described bacteria so far. Its versatile metabolism has allowed to colonize diverse and antagonist ecosystems by different members of this group. They are fundamentally aerobics and chemoorganotrophic bacteria, with oxidative metabolism. Its vegetative development generates sporulate ramified mycelia, the reason why *Streptomyce*s was considered a transition group between fungi and bacteria (Miyadoh, 1997).

Classification of *Streptomyces* has evolved in the past 100 years starting with the former and shallower morphological study of the substrate and aerial mycelia (Waksman, 1919), achieving later a deeper morphological analysis carried out by the International *Streptomyces* Project ISP (Shirling and Gottlieb, 1966), as well as the inclusion of chemotaxonomic, genetic, and more recently, genomic analyses. Amplification of the 16S rRNA gene is considered the first analysis for genetic analysis (Weisburg et al., 1991), but it is the multi-locus sequence analysis (MLSA), which provides a better resolution to species level relatedness (Guo et al., 2008; Rong and Huang, 2014). The development of platforms for next-generation sequencing has boosted the sequencing of whole genomes allowing the comparison of relative genomes that determine the degree of differentiation among compared genomes. Thus, pairwise genome comparison appeared as a robust method to replace the DNA–DNA hybridization method (Richter and Rosselló-Móra, 2009).

The genus *Streptomyces* is a well-known secondary metabolite producer. In fact, around 80% of current antibiotics are originally obtained from species of *Streptomyces* (Mast and Stegmann, 2018), and also some of them have the capacity to synthesize anti-inflammatory and antitumoral compounds (Barka et al., 2016). Former antibiotics were discovered from soil microorganisms, but new environments have been explored in the last years, as is the case of marine and subterranean environments (Gould, 2016; Tortorella et al., 2018; Rangseekaew and Pathom-Aree, 2019). *In silico* analysis focused on resistome and secondary metabolism mechanisms has been presented as a reliable tool, not only to discover new molecules and differentiate mechanisms involved in drug resistance but also to differentiate species of bacteria with identical 16S rRNA gene sequences (Antony-Babu et al., 2017) as is the case of *Streptomyces*, as well as to identify conserved specialized metabolites, supporting phylogenetic relationships (Vicente et al., 2018).

In this study, two strains, namely, MZ03-37^T^ and MZ03-48, were isolated from a lava tube (volcanic cave) located on La Palma Island (Canary Islands, Spain). The taxonomic position of the isolates was clarified using a polyphasic approach, which lead to the identification of a new species of the genus *Streptomyces*.

## Materials and Methods

### Bacterial Isolation and Culture Conditions

Strains MZ03-37^T^ and MZ03-48 were isolated on nutrient agar (NA, BD, Sparks, USA) with 3% of magnesium sulfate and 2% of glycerol at 28°C for 21 days, from two different locations in the lava tube Fuente de la Canaria (latitude: 28°35'25.2“N and longitude: 17°48′01.3”W) located in La Palma Island, Canary Islands (Spain) (Gonzalez-Pimentel et al., 2021). MZ03-37^T^ was isolated from a light-brown mucolite and MZ03-48 from a dark-brown microbial mat, both sampling sites separated by a distance of 15 m. Morphological, chemotaxonomical, and physiological studies were carried out from cultures on yeast extract-malt extract agar (International *Streptomyces* Project medium no 2, ISP2) at 28°C, except when otherwise indicated.

### Phylogenetic and Genome Annotation Analysis

Genomic DNA was extracted by implementing the Marmur method (Marmur, 1961). Amplification of the 16S rRNA gene was carried out using the method described by Laiz et al. (2009). Identification of the closest bacteria was determined using the global alignment algorithm on the EzBioCloud (Yoon et al., 2017). Additionally, the study of MLSA based on five housekeeping genes was carried out: *atpD* (ATP synthase β-subunit), *gyrB* (DNA gyrase β-subunit), *recA* (recombinase A), *rpoB* (RNA polymerase β-subunit), and *trpB* (tryptophan synthase β-subunit). The primers for amplification, PCR conditions, and sequencing of *atpD, recA, rpoB*, and *trpB* genes were described previously by Guo et al. (2008), whereas the primers for *gyrB* were redesigned by Rong et al. (2009). PCR products were sequenced by Macrogen (Seoul, Korea). The identification of closest-related strains was obtained through the BLAST algorithm (Altschul et al., 1990), in the GenBank database by the National Center for Biotechnology Information and the *Streptomyces* MLST database (http://pubmlst.org/streptomyces). Sequences of each gene and the closest-related strains were multiply aligned using MUSCLE (Edgar, 2004). Phylogenetic trees based on concatenated sequences of six housekeeping genes (16S rRNA-*atpD-gyrB-recA-rpoB-trpB*) were constructed using the maximum-likelihood (Felsenstein, 1981), neighbor-joining (Saitou and Nei, 1987), and maximum-parsimony (Kluge and Farris, 1969) methods in MEGA version 6 (Tamura et al., 2013). The tree robustness was evaluated using a bootstrap analysis of 1,000 resamplings.

The company MicrobesNG (Birmingham, UK) was responsible for sequencing genomic DNA in order to assemble the draft genome for both bacteria. Sequencing of genomic DNA of strains MZ03-37^T^, MZ03-48, and *Streptomyces palmae* CMU-AB204^T^ and subsequent assembly was described by Gonzalez-Pimentel et al. (2021). The genome of *Streptomyces ramulosus* NRRL B-2714^T^ was assembled following the same procedure using the raw data (Ju et al., 2015).

Functional annotation analyses were carried out focusing on the predicted genes by Prokka (Seemann, 2014) and the contigs assembled from sequenced genomes aimed at characterizing the function of genes and predicting the mechanisms for antimicrobial resistance, also called resistome, as well as the gene clusters involved in the secondary metabolism developed in every bacterium. The tools used for these purposes were Sma3s (Muñoz-Mérida et al., 2014) for functional annotations using the UniProt bacteria database with “uniprot” option, and antiSMASH in strict search mode (Blin et al., 2021) for secondary metabolite biosynthesis gene clusters prediction. Pairwise genome comparison between genomes of MZ03-37^T^ and MZ03-48 and *S. palmae* CMU-AB204^T^, *S. ramulosus* NRRL B-2714^T^, and *Streptomyces catenulae* NRRL B-2342^T^ was assessed calculating the average nucleotide identity (ANI) by means of BLAST+ (ANIb) (Camacho et al., 2008), MUMmer (ANIm) (Kurtz et al., 2004), as well as tetra-nucleotide signature algorithms through JspeciesWS web service (Richter et al., 2016) and orthoANI using EzBioCloud database (Yoon et al., 2017). ANIb, ANIm, and orthoANI establish a 95% threshold under which compared genomes belong to different bacteria, whereas the values above would suggest that compared genomes would belong to strains from the same species. TETRA is based on standardized tetrameric frequencies represented in “*z*-score” values, so fix three values to suggest genomes belong to the same (above 0.999) or different (below 0.989). When obtained *z*-score resulted between these two values, only ANIb, ANIm, and orthoANI values will be considered.

Mauve software for genome segment alignment (Darling et al., 2004) was used to analyze conserved gene clusters involved in curamycin production. R package-based visualization tools were used. Genes involved in pathways and biological processes were plotted by means of the “ggplot2” library, secondary metabolite prediction was represented through a heat map using the “gplots” library, and the “genoPlotR” library was used for genome segment comparison from MAUVE alignment.

The GenBank/EMBL/DDBJ accession numbers for MZ03-37^T^, MZ03-48, *S. palmae* CMU-AB204^T^, and *S. catenulae* NRRL B-2342^T^ are VKJP00000000, VKLS00000000, SRID00000000, and JODY00000000, respectively. *S. ramulosus* NRRL B-2714^T^ draft genome was assembled from the SRA file with accession number SRR7783857, following the same methodology for genome assembly used in this study.

### Phenotypic, Morphological, and Chemotaxonomic Features

Comparative studies were carried out in triplicate on ISP2 medium at 28°C for all strains. Spores were observed using light microscopy after 7 days of incubation. International *Streptomyces* Project medium no. 3 (Oatmeal agar), 4 (Inorganic salt starch agar), 5 (Glycerol asparagine agar), 6 (Peptone yeast Iron agar), and 7 (Tyrosine agar) were additionally used for morphological and physiological analyses. Oxidase activity was tested using BBL^TM^DrySlide^TM^ Oxidase (BD, Sparks, USA). The temperature range for growth was assessed at 5°C, 10°C, 20°C, 25°C, 28°C, 30°C, 32°C, 37°C, and 40°C. Salt tolerance was tested in the presence of 0–15% (w/v) NaCl with increases of 1% on nutrient agar (BD, Sparks, USA). Growth at different pH values was determined on trypticase soy broth and agar plates adjusted to pH 4.0–12.0 (at intervals of 1.0 pH unit) using HCl 1 M and NaOH 1 M solutions. The pH values were verified after autoclaving. Physiological characteristics were determined with API 20NE gallery (bioMérieux, Marcy l'Etoile, France), according to the manufacturer's instructions. The use of sugars as sole carbon sources was checked in a minimal medium containing M9 salts (Savic et al., 2007) and a 1% carbon source (w/v) (Miller, 1992). Cellular fatty acid profiles were analyzed in triplicate after collecting biomass from a culture grown for 3 days on TSA at 30°C following the methodology described by Jurado et al. (2009). Analysis of respiratory quinones and polar lipid composition were carried out by the Deutsche Sammlung von Mikroorganismen und Zellkulturen GmbH (Braunschweig, Germany).

## Results and Discussion

### Phylogenetic, Morphological, and Physiological Analyses

**Figure 1:**
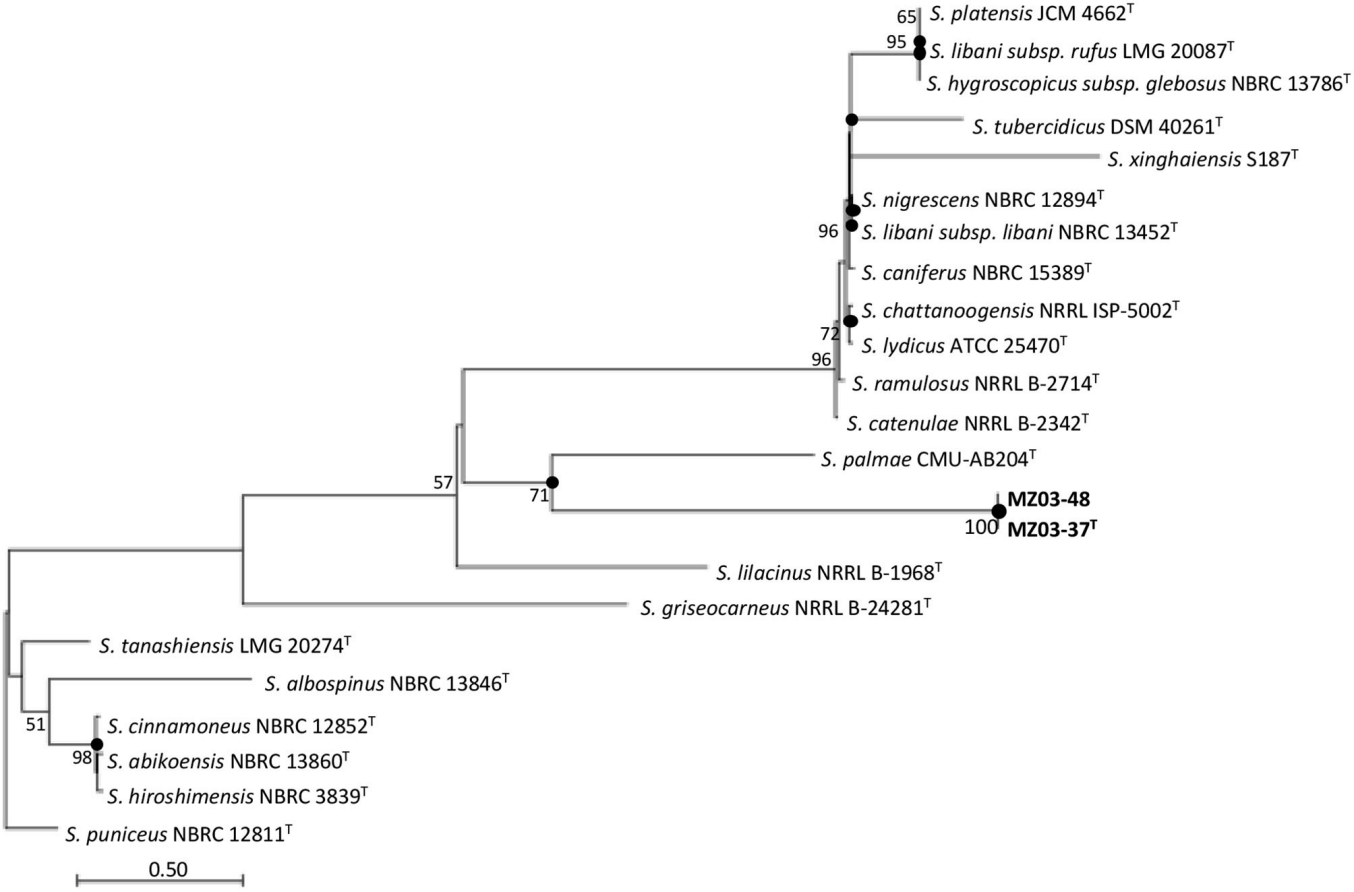
Maximum-likelihood trees based on six-gene concatenated sequences (16S rRNA-atpD-gyrB-recA-rpoB-trpB) showing the relationship of strains MZ03-37T and MZ03-48 and other species of the genus *Streptomyces*. Bootstrap values (N50%) are expressed as percentages of 1,000 replicates. Closed circles show the nodes that were also recovered by the neighbor-joining and maximum-parsimony algorithms. *Streptomyces puniceus* NBRC 12811T was used as the outgroup. Bar, 0.50 substitutions per nucleotide position.

The 16S rRNA gene sequence analysis revealed that strains MZ03-37^T^ and MZ03-48 were 100% identical among them and most closely related to *S. palmae* CMU-AB204^T^ (98.70%), *S. ramulosus* NRRL B-2714^T^ (98.28%), and *S. catenulae* NRRL B-2342^T^ (98.28%), equal or below the threshold suggested by Yarza et al. (2014) for identifying a new species of bacterium. MLSA analysis showed that *S. palmae* CMU-AB204^T^ (16S rRNA gene), *S. catenulae* NRRL B-2342^T^ (*atpD, gyrB, recA*, and *trpB* genes), and *S. ramulosus* (*rpoB* gene) were the closest relatives to MZ03-37^T^ and MZ03-48. The maximum-likelihood analysis based on concatenated housekeeping genes (Figure 1) showed that the closest relative of both strains was *S. palmae* CMU-AB204^T^ in a group well supported by a 71% bootstrap value. The species *S. catenulae* NRRL B-2342^T^ and *S. ramulosus* NRRL B-2714^T^ were grouped in a reliable group (98% bootstrap value), phylogenetically nearest to other *Streptomyces* species. These three species were selected as reference strains.

Strains MZ03-37^T^ and MZ03-48 were aerobic and Gram-positive. Cell morphology after 7 days of incubation at 28°C in ISP2 liquid medium showed well developed and abundant hyphae as described for species affiliated to the genus *Streptomyces* (Kämpfer, 2012). Both strains grew at 37°C and up to 15% of NaCl, but also these strains showed differences between each other and with respect to the reference strains (Table 1). They differed in their ability to grow at different pH and in the use of arginine as a source of carbon and energy. No significant differences were observed in the use of sole sources of carbon between MZ03-37^T^ and MZ03-48 beyond a weak growth using distinct sugars or sugar alcohols. Using *myo*-inositol and mannitol, MZ03-37^T^ grew well, whereas MZ03-48 showed a low development of biomass. These two strains seemed to present more characteristics in common with *S. catenulae* and *S. ramulosus* rather than *S. palmae*. These five bacteria showed GC contents between 72.1 and 73%, in accordance with the GC-rich content of *Streptomyces* species.

**Table 1:** Differential characteristics of strains MZ03-37^T^, MZ03-48, and related species.

**Test**	**1**	**2**	**3**	**4**	**5**
NaCl concentration for growth:					
6 % (w/v)	+	+	–	+	+
Optimal pH for growth	6–9	5–10	6–9	5–10	5–10
Enzymatic activity:					
Urease	+	+	–	+	+
Arginine dihydrolase	+	–	–	+	–
β-glucosidase	+	+	+	–	+
β-galactosidase	–	–	+	–	+
Oxidase	–	–	+	–	–
Assimilation of:					
Arabinose	–	–	+	–	–
Mannose	+	+	–	+	+
D-mannitol	+	+	–	+	+
D-maltose	–	–	–	+	+
Adipic acid	–	–	+	–	+
Trisodium citrate	+	+	–	–	–
Nitrate reduction	–	–	+	+	–
Growth with sole carbon source (1% p/v):					
Xylose	+	+	–	(+)	+
Mannitol	+	(+)	–	+	+
Diffusible pigment	Not	Not	Not	Dark	Dark
%GC content	72.1	72.1	72.4	73	72.7

*Strains: 1, MZ03-37^T^; 2, MZ03-48; 3, Streptomyces palmae CMU-AB204^T^; 4, Streptomyces catenulae NRRL B-2342^T^; 5, Streptomyces ramulosus NRRL B-2714^T^. +, positive; (+) weak positive; –, negative*.

Results from the International *Streptomyces* Project medium showed notable differences between the study strains. MZ03-37^T^ presented a light brown substrate mycelium and a white-colored aerial mycelium in ISP2 and ISP3, mustard tan in ISP5, beige in ISP6, and brown for both mycelia in IPS4 and ISP7, whereas MZ03-48 developed a light-black colored substrate and aerial mycelia in ISP2, ISP3, and ISP7 (Supplementary Figure S1) and a light-orange for both mycelia in ISP5, ISP6, and ISP4, but with white aerial mycelium in the last one (Supplementary Table S1). None of the analyzed strains produced soluble pigment on any of the tested ISP media. *Streptomyces* MZ03-37^T^ and MZ03-48 were also compared with the type strains grown in the same culture medium where the study bacteria were isolated, NA with 3% magnesium sulfate, and 2% glycerol (Supplementary Figure S1). MZ03-37^T^ formed a white aerial mycelium and a light brown substratum, MZ03-48 developed a light black substratum and aerial mycelium, *S. catenulae* formed a beige substratum and an aerial mycelium, *S. ramulosus* presented an aerial mycelium white-colored and a beige substrate, while *S. palmae* did not grow on this medium.

### Pairwise Genome Comparison

The strains MZ03-37^T^ and MZ03-48 (Table 2) showed ANIb and ANIm, as well as OrthoANI values above the 99.9% among them, and less than 93.11% with *S. ramulosus*, the closest species observed in this analysis. Moreover, the results of TETRA calculations showed a high coefficient (>0.999) between the strains MZ03-37^T^ and MZ03-48 and a low coefficient (<0.999) between any of the closest species and both strains supporting the species circumscription. These values suggest that strains MZ03-37^T^ and MZ03-48 belonged to the same species since they showed high similarity after using referenced algorithms, whereas results obtained against the closest species point out that they could be considered as a new species within the genus *Streptomyces*.

**Table 2:** Values for pairwise genome comparison with ANIb, ANIm, orthoANI (%), and TETRA (0–1).

	**1**	**2**	**3**	**4**	**5**
**ANIb**					
**1**	–	76.95	77.82	76.98	77.01
**2**	77.97	–	92.06	91.32	91.35
**3**	77.18	92.40	–	91.69	91.69
**4**	77.98	91.20	91.59	–	99.96
**5**	77.93	91.14	91.62	99.98	–
**ANIm**					
**1**	–	85.52	85.62	85.54	85.56
**2**	85.52	–	93.36	92.57	92.65
**3**	85.64	93.37	–	93.05	93.11
**4**	85.54	92.56	93.05	–	99.99
**5**	85.55	92.65	93.11	99.99	–
**TETRA**					
**1**	–	0.93579	0.95677	0.95066	0.95231
**2**	0.93579	–	0.99299	0.99246	0.99379
**3**	0.95677	0.99299	–	0.99534	0.99664
**4**	0.95066	0.99246	0.99534	–	0.99949
**5**	0.95231	0.99379	0.99664	0.99949	–
**OrthoANI**					
**1**	–	–	–	–	–
**2**	78.72	–	–	–	–
**3**	79.15	92.66	–	–	–
**4**	79.14	91.67	92.23	–	–
**5**	79.24	91.91	92.30	99.96	–

*Strains: 1, Streptomyces palmae CMU-AB204^T^; 2, Streptomyces catenulae NRRL B-2342^T^; 3, Streptomyces ramulosus NRRL B-2714^T^; 4, MZ03-37^T^; 5, MZ03-48. –, Means that pairwise genome comparison against themselves result in 100% identity*.

#### *In silico* Analyses for Resistome and Secondary Metabolism

Genome characteristics are described in Supplementary Table S2. Sma3s resulted in 1,562 and 1,565 annotations from 6,336 and 6,410 queried sequences using UniProt – SwissProt curated database, for strains MZ03-37^T^ and MZ03-48, respectively. Genes predicted to a role in antimicrobial biosynthesis were the most abundant after amino acid and cofactor biosynthesis, with 65 out of a total of 652 genes predicted on pathway annotations, in MZ03-37^T^, and 67 out of 654 in MZ03-48. In addition, genes involved in antibiotic biosynthesis and antibiotic resistance were significantly abundant within the biological process category, with 83 and 44 out of 1,500 annotated genes, respectively, in MZ03-37^T^, and 82 and 42 out of 1,500 annotated sequences predicted in MZ03-48 (Supplementary Figure S2).

Predicted genes involved in antimicrobial mechanisms for resistance and biosynthesis in MZ03-37^T^ and MZ03-48 strains using Sma3s and UniProt-SwissProt database emphasize *Streptomyces* members as a biological reservoir for bioactive compounds, both described and not discovered yet (Supplementary Table S3). Genes or gene clusters associated with linocin-M18 (Valdés-Stauber and Scherer, 1994) and curamycin (Bergh and Uhlén, 1992) were predicted with a 50%-75% similarity in both strains, MZ03-37^T^ and MZ03-48. Monensin gene cluster (Arrowsmith et al., 1992) was predicted to get more than a 75% similarity with every aligned gene only in strain MZ03-37^T^. Likewise, several annotated sequences exhibited descriptive information on antimicrobial resistance mechanisms related to transport and/or efflux systems as well as enzymes used to degrade the antimicrobials. Thus, more than 50% of sequence similarity genes were involved in resistance against cationic antimicrobial peptides (CAMPs), macrolides, β-lactam, nosiheptide, among others. With a similarity of 75% or higher, resistance for chloramphenicol and rifampicin was predicted in both strains. Also, new possible molecules not described yet could be produced by MZ03-37^T^ and MZ03-48 since there were predicted sequences showing high similarity with described genes which encoded to polyketide antibiotics as was the case of the putative polyketide hydroxylase (*schC*) (Blanco et al., 1993a,b) with a 69% of similarity, as well as other antibiotic-related synthases and transferases that appear in non-completed pathways in MZ03-37^T^ and MZ03-48 strains.

### Secondary Metabolite Comparison

Beyond antimicrobial production, the genus *Streptomyces* is well known for including species being able to produce additional bioactive compounds with extensible uses in biotechnology (Goodfellow and Fiedler, 2010). The antiSMASH web tool predicted a total of 28 gene clusters involved in secondary metabolism activity in MZ03-37^T^ and 29 gene clusters in MZ03-48 (Supplementary Table S4). Both strains shared the same number of types of metabolites, with the exception of non-ribosomal peptide synthases (NRPS), having one more predicted gene cluster in MZ03-48. In summary, for MZ03-37^T^, eight NRPS and nine for MZ03-48, four terpenes, four siderophores, three type 1 polyketide synthases (T1PKS), two type 2 polyketide synthases (T2PKS), one type 3 polyketide synthases (T3PKS), two butyrolactone, two bacteriocins, one ectoine, and one lanthipeptide were predicted.

**Figure 2:**
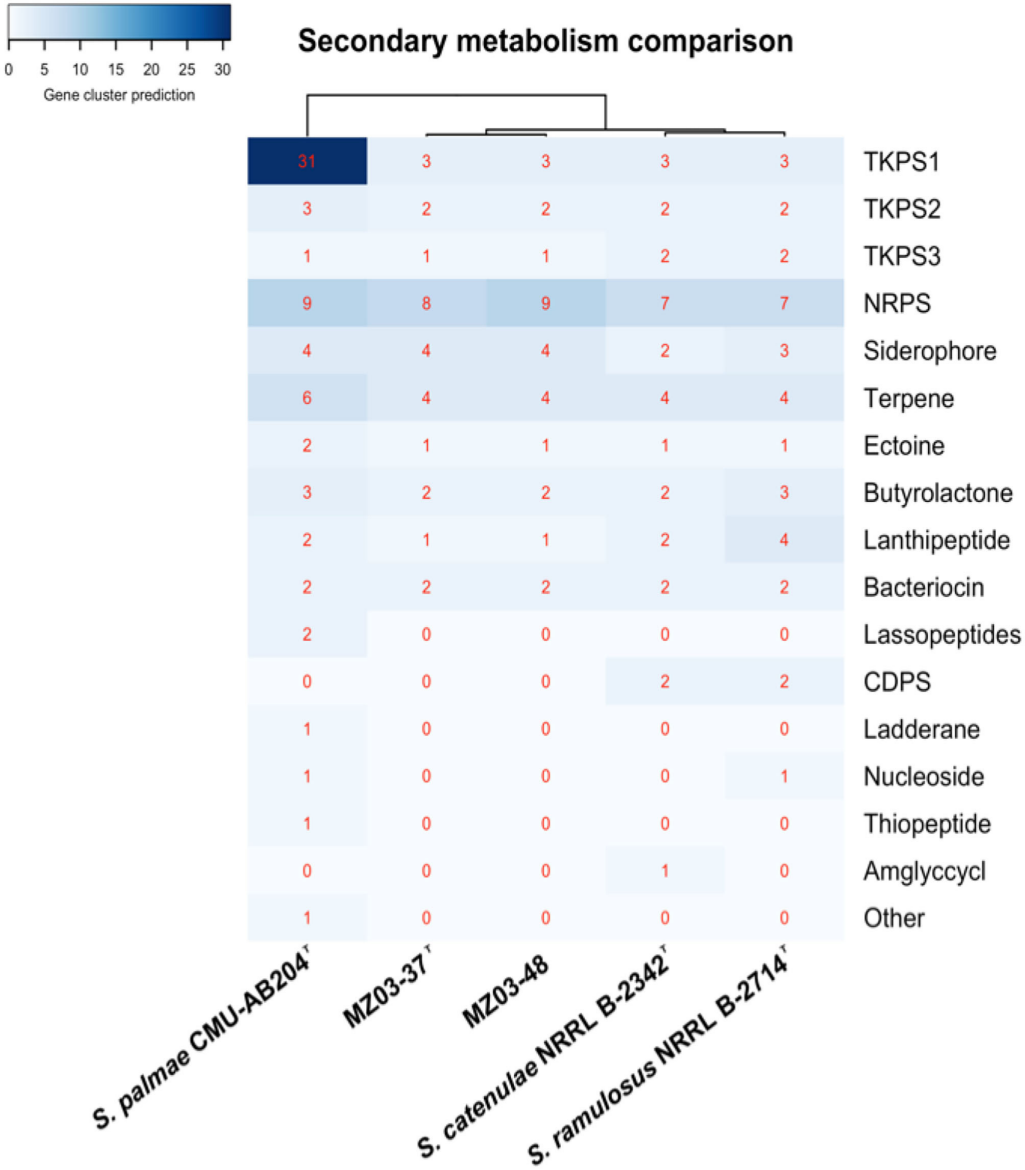
Secondary metabolism comparison using anti-SMASH. Abundance of secondary metabolites predicted. TKPS1, type 1 polyketides synthase; TKPS2, type 2 polyketides synthase; TKPS3, type 3 polyketides synthase; NRPS, nonribosomal peptide synthase; CDPS, tRNA-dependent cyclodipeptide synthase; Amglyccycl, aminoglycoside/aminocyclitol cluster; other, cluster containing a secondary metabolite-related protein that does not fit into any other category. Cladogram built using Euclidean algorithm.

Likewise, additional anti-SMASH analyses were implemented for type strains for secondary metabolism comparison (Figure 2). Sixty-nine gene clusters were predicted for *S. palmae* which presented the most active secondary metabolism. Notably, 34 gene clusters were predicted for *S. ramulosus* and 30 for *S. catenulae*. Shared metabolites on the five strains were scarce, being present some typical molecules already described before for *Streptomyces* (Vicente et al., 2018), as it was the case of geosmin (Gerber and Lechevalier, 1965), hopene (Poralla et al., 2000), and ectoine (Malin and Lapidot, 1996), as well as the antibiotic curamycin (Bergh and Uhlén, 1992).

**Figure 3:**
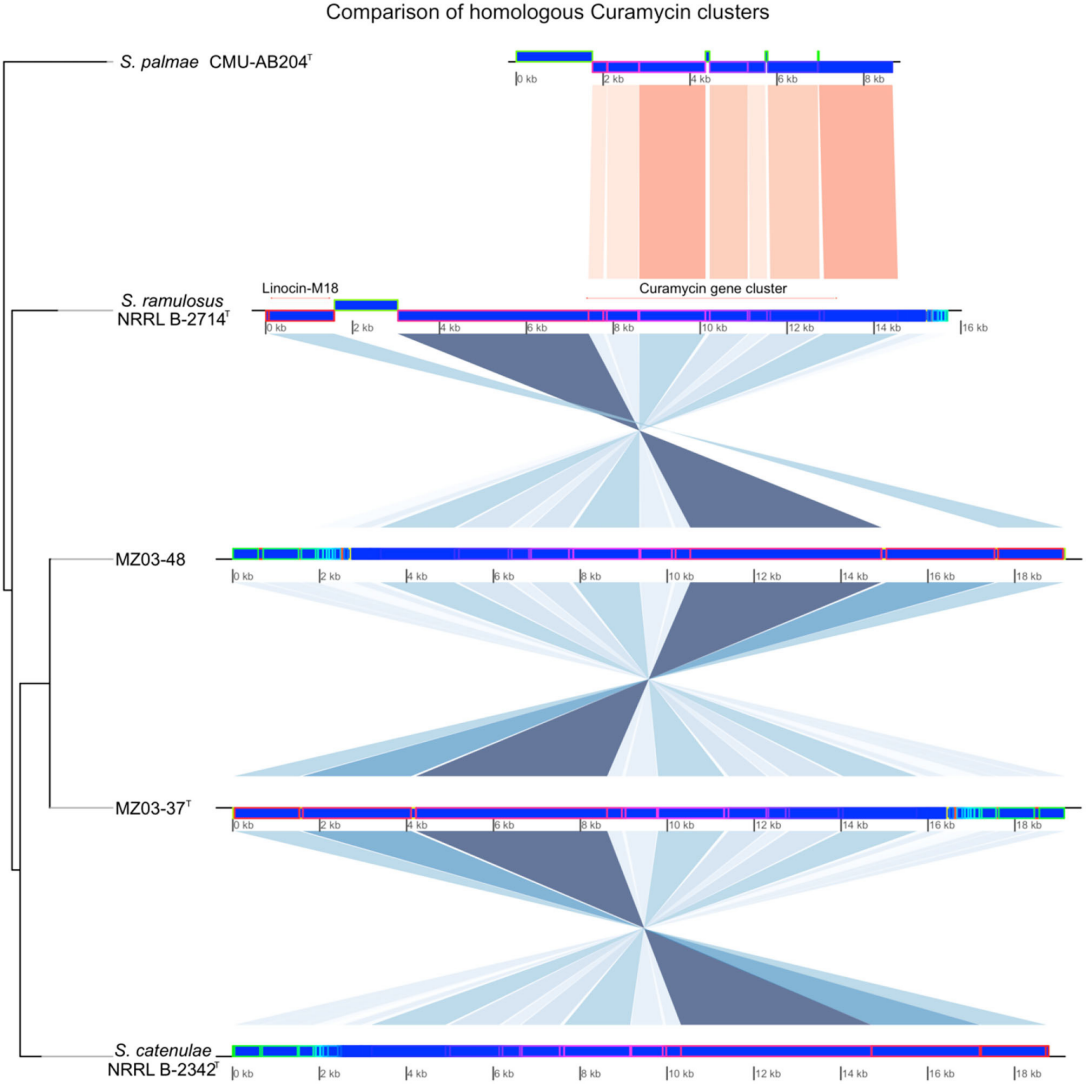
Comparison of segments where Curamycin-Linocin-M18 gene clusters were predicted. Newick phylogenetic tree using Euclidean algorithm was built to determine the relatedness among contigs of the five bacteria. Conserved regions identified by MAUVE were represented with shades of blue and red.

Naringenin gene cluster was also predicted for MZ03-48, *S. catenulae*, and *S. ramulosus*, missing in MZ03-37^T^ and *S. palmae*. Naringenin, along with the additional NRPS cluster predicted in MZ03-48, was supposed for the main difference between this strain and MZ03-37^T^. Linocin-M18 was predicted for all analyzed strains with the exception of *S. palmae*. Linocin-M18 was located along with curamycin gene cluster in the same contig (Figure 3). BLAST alignment resulted in a 100% of identity in the case of strains MZ03-37^T^ and predicted in reverse orientation, MZ03-48. These two contigs aligned a 94.03% with the contig from *S. catenulae*, a 93.82% with *S. ramulosus*, and a 78.31% was aligned against contig where curamycin gene cluster was predicted for *S. palmae*. No Linocin-M18 gene cluster was predicted in other assembled contigs for this last bacterium.

Comparison of predicted secondary metabolites could add a new insight for differing closely related bacteria, as is the case of *Streptomyces* spp., beyond the discovery of new metabolites from microorganisms dwelling in specific environments, as is the case of caves and lava tubes (Vicente et al., 2018; Sottorff et al., 2019). Differences in the prediction of gene clusters, as well as similarities in particular segments to compare, showed a trend for bacterial relatedness which reinforces the results from the polyphasic characterization, multilocus sequence typing, and full genome sequencing analyses.

Based on all characteristics, strains MZ03-37^T^ and MZ03-48 represent a new species, for which we proposed the name *Streptomyces benahoarensis* sp. nov.

### Description of *S. benahoarensis* sp. nov.

*Streptomyces benahoarensis* (be.na.hoar.en'sis. N.L. fem. adj. *benahoarensis*, originating from Benahoare, the Guanche name of La Palma, the island where both strains were isolated) is Gram-positive and aerobic, and forms rectiflexibiles spore chains. No fragmentation is developed neither aerial nor substrate mycelia. Growth was observed at 10–37°C (optimal 28–32°C), with 0–15% (w/v) NaCl (optimal 0–10%), and at pH 4.0–10.0 (optimal 6–9) for MZ03-37^T^. It is oxidase-negative. Nitrate is not reduced to nitrite. Indole is produced from tryptophan, and glucose fermentation does not occur. It is positive for arginine dihydrolase, urease, β-glucosidase, and protease and negative for β-galactosidase. It shows positive assimilation for glucose, mannose, mannitol, N-acetyl-glucosamine, potassium gluconate, malate, trisodium citrate, and phenylacetic acid and negative assimilation for arabinose, maltose, capric acid, and adipic acid. It uses sacarose, maltose, mannose, glycerol, xylose, *myo*-inositol, and mannitol as carbon sources for growth but not dextran. It shows weak growth with D-galactose, lactose, fructose, and glucose, and good growth on ISP media (2, 3, 4, 5, 6, and 7). Soluble pigments are not produced, and melanin is not formed. Major cellular fatty acids are iso-C_16:0_, anteiso-C_15:0_, C_16:0_, and iso-C_14:0_. The cell wall contains LL-diaminopimelic acid in its peptidoglycan. The GC content of the type strain is 72.1 mol%.

The type strain, MZ03-37^T^ (=CECT 9805^T^ = DSMZ 8002^T^), was isolated from a lava tube speleothem collected in Fuente de la Canaria (La Palma Island, Canary Islands, Spain); a reference strain, MZ03-48 (= CECT 9806 = DSMZ 8011) was isolated from a microbial mat from Fuente de la Canaria lava tube (La Palma Island, Canary Islands, Spain).

## Data Availability Statement

The datasets presented in this study can be found in online repositories. The names of the repository/repositories and accession number(s) can be found in the article/Supplementary Material.

## Author Contributions

JG-P developed the ideas and designed the experimental plans. CS-J supervised the research and provided funding support. JG-P and VJ performed experiments. JG-P, VJ, and BH analyzed the data. JG-P, VJ, and CS-J prepared the manuscript. All authors contributed to this study and approved the submitted version.

## Funding

This study was supported by the project 0483_PROBIOMA_5_E, co-financed by the European Regional Development Fund within the framework of the Interreg V A Spain – Portugal program (POCTEP) 2014–2020. 2015 and 2016 field trips to the cave were supported by a former Spanish Ministry of Economy, Industry, and Competitiveness (MINEICO) project CGL2013-41674-P.

## Conflict of Interest

The authors declare that the research was conducted in the absence of any commercial or financial relationships that could be construed as a potential conflict of interest.

## Publisher's Note

All claims expressed in this article are solely those of the authors and do not necessarily represent those of their affiliated organizations, or those of the publisher, the editors and the reviewers. Any product that may be evaluated in this article, or claim that may be made by its manufacturer, is not guaranteed or endorsed by the publisher.

## Supplementary Material

Supplementary Figure S1 Morphology of colonies MZ03-48 (A), MZ03-37^T^ (B), and reference strains *Streptomyces ramulosus* NRRL B-2714^T^ (C) and *Streptomyces catenulae* NRRL B-2342^T^ (D). Plates cultured on nutrient agar (NA, BD, Sparks, USA) with 3% of magnesium sulfate and 2% of glycerol at 28°C for 7 days.

Supplementary Figure S2 Genes classified by functional categories in pathways (A) and biological processes (B) from curated UniProt-SwissProt. “Others” gathered those categories with less than 1% of total genes.

Supplementary Table S1 Morphology and physiology observed after culturing of MZ03-37^T^ and MZ03-48 in International *Streptomyces* Project media.

Supplementary Table S2 Genome characterization of *Streptomyces* strains MZ03-37^T^, MZ03-48, and reference species. rRNA, ribosomal RNA; tRNA, transfer RNA; tmRNA, transfer-messenger RNA.

Supplementary Table S3 Antimicrobial biosynthesis and resistance mechanisms identified in MZ03-37^T^ and MZ03-48 by Sma3s. The table differentiates identifications with similarities from 50 to 75% and from 75 to 100%. nonavailable (N/A) gene names are replaced by their protein names: (A) monensin polyketide synthase ACP; monensin polyketide synthase putative ketoacyl reductase; putative polyketide beta-ketoacyl synthase 1; putative polyketide beta-ketoacyl synthase 2; granaticin polyketide synthase bifunctional cyclase/dehydratase. (B) Putative nosiheptide resistance regulatory protein. (1) Cationic antimicrobial Peptides. (2) Tetracyclines, macrolides, lincosamides, and aminoglycosides (Morris et al., 2005). (3) Tylosin and erythromycin (Quirós et al., 1998). (4) Only present in MZ03-37^T^.

Supplementary Table S4 Secondary metabolites predicted for MZ03-37^T^ and MZ03-48 by anti-SMASH. Gene clusters predicted without reference to the identified closest cluster in the database were omitted. “Similarity” pointed out the % of genes predicted that have been identified in described biosynthetic clusters. The minimum threshold used by anti-SMASH for finding homology fixed a BLAST e-value below 1E-05, a 30% minimal sequence identity, and a coverage of >25% of the sequence. ^1^The query sequence covered only 25% of the closest sequence.
