# Supplementary material for: Streptomyces benahoarensis sp. nov. Isolated From a Lava Tube of La Palma, Canary Islands, Spain
Source: Front Microbiol. 2022 May 16;13:907816. doi: 10.3389/fmicb.2022.907816 (PMC9149447; doi:10.3389/fmicb.2022.907816)
Supplement: Supplementary Table S1 — Morphology and physiology observed after culturing of MZ03-37T and MZ03-48 in International Streptomyces Project media. [file Data_Sheet_1.zip › Table S1.DOCX]

**TABLE S1.** Morphology and physiology observed after culturing of MZ03-37^T^ and MZ03-48 in International *Streptomyces* Project media.

| **Culture medium** | **Strain** | **Growth** | **Aerial mycelium color** | **Substrate mycelium color** |
| --- | --- | --- | --- | --- |
| ISP2 | MZ03-37^T^ | Good | White | Light-brown |
| ISP3 |  |  | White | Light-brown |
| ISP4 |  |  | Brown | Brown |
| ISP5 |  |  | Mustard tan | Mustard tan |
| ISP6 |  |  | Beige | Beige |
| ISP7 |  |  | Brown | Brown |
| ISP2 | MZ03-48 | Good | Light-black | Light-black |
| ISP3 |  |  | Light-black | Light-black |
| ISP4 |  |  | Light-orange | White |
| ISP5 |  |  | Light-orange | Light-orange |
| ISP6 |  |  | Light-orange | Light-orange |
| ISP7 |  |  | Light-black | Light-black |
