# Supplementary material for: Streptomyces benahoarensis sp. nov. Isolated From a Lava Tube of La Palma, Canary Islands, Spain
Source: Front Microbiol. 2022 May 16;13:907816. doi: 10.3389/fmicb.2022.907816 (PMC9149447; doi:10.3389/fmicb.2022.907816)
Supplement: Supplementary Table S1 — Morphology and physiology observed after culturing of MZ03-37T and MZ03-48 in International Streptomyces Project media. [file Data_Sheet_1.zip › Table S2.DOCX]

**TABLE S2.** Genome characterization of Streptomyces strains MZ03-37T, MZ03-48 and reference species.

| **Strain** | **Genome size** | **GC cont. (%)** | **Sequences** | **rRNA** | **tRNA** | **tmRNA** |
| --- | --- | --- | --- | --- | --- | --- |
| MZ03-37^T^ | 6,995,890 | 72.2 | 6075 | 9 | 80 | 1 |
| MZ03-48 | 6,916,377 | 72.1 | 6019 | 9 | 79 | 1 |
| *S. palmae* | 7,497,959 | 72.5 | 6377 | 10 | 84 | 1 |
| *S. catenulae* | 7,046,907 | 73.0 | 6023 | 4 | 84 | 1 |
| *S. ramulosus* | 7,504,332 | 72.7 | 6517 | 11 | 90 | 1 |

rRNA: Ribosomal RNA; tRNA: Transfer RNA; tmRNA: Transfer-messenger RNA.
