# Supplementary material for: Streptomyces benahoarensis sp. nov. Isolated From a Lava Tube of La Palma, Canary Islands, Spain
Source: Front Microbiol. 2022 May 16;13:907816. doi: 10.3389/fmicb.2022.907816 (PMC9149447; doi:10.3389/fmicb.2022.907816)
Supplement: Supplementary Table S1 — Morphology and physiology observed after culturing of MZ03-37T and MZ03-48 in International Streptomyces Project media. [file Data_Sheet_1.zip › Table S3.DOCX]

**Table S3.** Antimicrobial biosynthesis and resistance mechanisms identified in MZ03-37^T^ and MZ03-48 by Sma3s.

|  | **>50% similarity** | **>75% similarity** | **Antibiotic** |
| --- | --- | --- | --- |
| **Biosynthesis** |  | *N/A* (5 genes)^A^ | Monensin |
|  | *lin* |  | Linocin-M18 |
|  | *curABCDEFG* |  | Curamycin |
|  | *dhbABCEF* |  | Bacillibactin |
| **Resistance** |  | *cmlR* | Chloramphenicol |
|  |  | *relK* | Rifampicim |
|  | *lysX* |  | CAMPs^1^ |
|  | *whiB7* |  | -^2^ |
|  | *oleD* |  | Macrolides^3^ |
|  | *pur8*^4^ |  | Puromycin |
|  | *tcmA* |  | Tetracenomycin C |
|  | *vgb* |  | Virginiamycin B |
|  | *ast1* |  | Beta-lactam |
|  | *N/A*^B^ |  | Nosiheptide |

The table differentiates identifications with similarities from 50% to 75% and from 75% to 100%. Non-available (N/A) gene names are replaced by their protein names: (A) Monensin polyketide synthase ACP; Monensin polyketide synthase putative ketoacyl reductase; Putative polyketide beta-ketoacyl synthase 1; Putative polyketide beta-ketoacyl synthase 2; Granaticin polyketide synthase bifunctional cyclase/dehydratase. (B) Putative nosiheptide resistance regulatory protein. (1) Cationic AntiMicrobial Peptides. (2) Tetracyclines, macrolides, lincosamides, and aminoglycosides (Morris et al., 2005). (3) Tylosin and erythromycin (Quirós et al., 1998). (4) Only present in MZ03-37^T^.
