# Supplementary material for: Streptomyces benahoarensis sp. nov. Isolated From a Lava Tube of La Palma, Canary Islands, Spain
Source: Front Microbiol. 2022 May 16;13:907816. doi: 10.3389/fmicb.2022.907816 (PMC9149447; doi:10.3389/fmicb.2022.907816)
Supplement: Supplementary Table S1 — Morphology and physiology observed after culturing of MZ03-37T and MZ03-48 in International Streptomyces Project media. [file Data_Sheet_1.zip › Table S4.DOCX]

**Table S4.** Secondary metabolites predicted for MZ03-37^T^ and MZ03-48 by antiSMASH.

| **Strain** | **Type** | **Closest cluster** | **Similarity (%)** |
| --- | --- | --- | --- |
| **MZ03-37^T^**  **MZ03-48** | NRPS | Bacillibactin | 100 |
|  |  | Stenothricin | 22 |
|  |  | Cinnamycin | 9 |
|  |  | Rhizomide A/B/C | 100^1^ |
|  | Terpene | Hopene | 61 |
|  |  | 2-methylisoborneol | 100 |
|  |  | Geosmin | 100 |
|  | Butyrolactone | Rabelomycin / Dehydrorabelomycin / Fluostatin F/G/H | 3 |
|  |  | Lactonamycin | 5 |
|  | T1PKS | C-1027 | 9 |
|  |  | ML-449 | 37 |
|  | T2PKS | Curamycin | 100 |
|  |  | Lugdunomycin | 48 |
|  | Bacteriocin | Linocin M18 | 100 |
|  | Ectoine | Ectoine | 100 |
| **MZ03-37^T^** | NRPS | Rimosamide | 14 |
| **MZ03-48** | T3PKS | Naringenin | 100 |

Gene clusters predicted without reference of identified closest cluster in database was omitted. ‘Similarity’ pointed out the % of genes predicted that have been identified in described biosynthetic clusters. Minimum threshold used by antiSMASH for finding homology fixed a BLAST e-value below 1E-05, a 30% minimal sequence identity and a coverage of >25% of the sequence. ^1^The query sequence covered only a 25% of closest sequence.
